# Supplementary material for: Hippo signaling differentially regulates distal progenitor subpopulations and their transitional states to construct the mammalian lungs
Source: Nat Commun. 2026 Apr 3;17:4802. doi: 10.1038/s41467-026-71253-x (PMC13219436; doi:10.1038/s41467-026-71253-x)
Supplement: Supplementary file 3 — Reporting Summary [file 41467_2026_71253_MOESM3_ESM.pdf]

Corresponding author(s): Pao-Tien Chuang

Last updated by author(s): Mar 7, 2026

Reporting Summary

Nature Portfolio wishes to improve the reproducibility of the work that we publish. This form provides structure for consistency and transparency in reporting. For further information on Nature Portfolio policies, see our Editorial Policies and the Editorial Policy Checklist.

Statistics

For all statistical analyses, confirm that the following items are present in the figure legend, table legend, main text, or Methods section.

n/a	Confirmed	
☐	☒	The exact sample size (*n*) for each experimental group/condition, given as a discrete number and unit of measurement
☐	☒	A statement on whether measurements were taken from distinct samples or whether the same sample was measured repeatedly
☐	☒	The statistical test(s) used AND whether they are one- or two-sided<br>*Only common tests should be described solely by name; describe more complex techniques in the Methods section.*
☒	☐	A description of all covariates tested
☒	☐	A description of any assumptions or corrections, such as tests of normality and adjustment for multiple comparisons
☐	☒	A full description of the statistical parameters including central tendency (e.g. means) or other basic estimates (e.g. regression coefficient) AND variation (e.g. standard deviation) or associated estimates of uncertainty (e.g. confidence intervals)
☐	☒	For null hypothesis testing, the test statistic (e.g. *F*, *t*, *r*) with confidence intervals, effect sizes, degrees of freedom and *P* value noted<br>*Give P values as exact values whenever suitable.*
☒	☐	For Bayesian analysis, information on the choice of priors and Markov chain Monte Carlo settings
☒	☐	For hierarchical and complex designs, identification of the appropriate level for tests and full reporting of outcomes
☐	☒	Estimates of effect sizes (e.g. Cohen's *d*, Pearson's *r*), indicating how they were calculated

Our web collection on statistics for biologists contains articles on many of the points above.

Software and code

Policy information about availability of computer code

Data collection	Confocal images were captured on a Leica SPE laser-scanning confocal microscope;<br>Whole-mount immunostaining and whole-lung imaging were captured on a Nikon Eclipse E1000 microscope with a SPOT 2.3 CCD camera;<br>Quantitative PCR was performed on an Applied Biosystems QuantStudioTM 5 Real-Time PCR System;<br>Library quality of bulk RNA-Seq was evaluated with an Agilent 2100 Bioanalyzer;<br>Library of bulk RNA-Seq was sequenced on BGISEQ-500 platform.<br>Measurement of the diameter, distance, thickness, area and the percentage of the cell were performed using ImageJ.
Data analysis	Confocal images adjustments (red/green/blue/grey histograms and channel merges) were performed using LAS AF Lite;<br>Adjustment of the color and lightness of the whole-mount immunostaining and whole-lung imaging pictures was performed using ImageJ;<br>EdU incorporation related quantification were performed using ImageJ;<br>The graph was generated using GraphPad Prism 7;<br>Differential gene expression, gene ontology (GO) enrichment analyses and the barplot of gene ontology enrichment were performed with RStudio, R version 3.4.0;<br>Heatmap images were generated using online Heatmapper software.<br>Data processing and analysis was performed using Cell Ranger version 8.0.1, mouse reference genome [refdata-gex-mm10-2020-A], R version 4.5.1 (2025-06-13), and PyScenic version 0.12.1. For multiomics, raw sequencing reads were processed using the 10x Genomics Cell Ranger Arc (v2.0.2) pipeline.<br>The following R packages were used:<br>arrow [v21.0.0]<br>batchelor [v1.24.0]

BSgenome.Mmusculus.UCSC.mm10 [v1.4.3]
CellRef [v0.1.0]
ChIPseeker [v1.44.0]
doParallel [v1.0.17]
DoubletFinder [v2.0.6]
dplyr [v1.1.4]
EnsDb.Mmusculus.v79 [v2.99.0]
foreach [v1.5.2]
future [v1.67.0]
future.apply [v1.20.0]
ggplot2 [v3.5.1]
ggraph [v2.2.2]
ggrepel [v0.9.6]
harmony [v1.2.3]
hdf5r [v1.3.12]
igraph [v2.1.4]
Matrix [v1.7.3]
Matrix.utils [v0.9.7]
MatrixGenerics [v1.21.0]
monocle3 [v1.3.7]
org.Mm.eg.db [v3.21.0]
patchwork [v1.3.1]
pcaMethods [v2.0.0]
presto [v1.0.0]
purrr [v1.1.0]
R.utils [v2.13.0]
RColorBrewer [v1.1.3]
RcppArmadillo [v14.6.0.1]
seqLogo [v1.74.0]
Seurat [v5.2.1]
SeuratDisk [v0.0.0.9021]
SeuratWrappers [v0.4.0]
Signac [v1.14.0]
SingleCellExperiment [v1.31.1]
SummarizedExperiment [v1.39.1]
tidyverse [v2.0.0]
TxDb.Mmusculus.UCSC.mm10.knownGene [v3.10.0]
velocyto.R [v0.6]

For manuscripts utilizing custom algorithms or software that are central to the research but not yet described in published literature, software must be made available to editors and reviewers. We strongly encourage code deposition in a community repository (e.g. GitHub). See the Nature Portfolio guidelines for submitting code & software for further information.

Data

Policy information about availability of data

All manuscripts must include a data availability statement. This statement should provide the following information, where applicable:

- Accession codes, unique identifiers, or web links for publicly available datasets
- A description of any restrictions on data availability
- For clinical datasets or third party data, please ensure that the statement adheres to our policy

Datasets have been deposited in the NCBI Gene Expression Omnibus (GEO) database and are accessible through the GEO Series (GSE) accession numbers GSE269537 and GSE319370.

Research involving human participants, their data, or biological material

Policy information about studies with human participants or human data. See also policy information about sex, gender (identity/presentation), and sexual orientation and race, ethnicity and racism.

Reporting on sex and gender	*Use the terms sex (biological attribute) and gender (shaped by social and cultural circumstances) carefully in order to avoid confusing both terms. Indicate if findings apply to only one sex or gender; describe whether sex and gender were considered in study design; whether sex and/or gender was determined based on self-reporting or assigned and methods used.* *Provide in the source data disaggregated sex and gender data, where this information has been collected, and if consent has been obtained for sharing of individual-level data; provide overall numbers in this Reporting Summary. Please state if this information has not been collected.* *Report sex- and gender-based analyses where performed, justify reasons for lack of sex- and gender-based analysis.*

Reporting on race, ethnicity, or other socially relevant groupings	*Please specify the socially constructed or socially relevant categorization variable(s) used in your manuscript and explain why they were used. Please note that such variables should not be used as proxies for other socially constructed/relevant variables (for example, race or ethnicity should not be used as a proxy for socioeconomic status).*<br>*Provide clear definitions of the relevant terms used, how they were provided (by the participants/respondents, the researchers, or third parties), and the method(s) used to classify people into the different categories (e.g. self-report, census or administrative data, social media data, etc.)*<br>*Please provide details about how you controlled for confounding variables in your analyses.*
Population characteristics	*Describe the covariate-relevant population characteristics of the human research participants (e.g. age, genotypic information, past and current diagnosis and treatment categories). If you filled out the behavioural & social sciences study design questions and have nothing to add here, write "See above."*
Recruitment	*Describe how participants were recruited. Outline any potential self-selection bias or other biases that may be present and how these are likely to impact results.*
Ethics oversight	*Identify the organization(s) that approved the study protocol.*

Note that full information on the approval of the study protocol must also be provided in the manuscript.

Field-specific reporting

Please select the one below that is the best fit for your research. If you are not sure, read the appropriate sections before making your selection.

[x] Life sciences [ ] Behavioural & social sciences [ ] Ecological, evolutionary & environmental sciences

For a reference copy of the document with all sections, see nature.com/documents/nr-reporting-summary-flat.pdf

Life sciences study design

All studies must disclose on these points even when the disclosure is negative.

Sample size	For all the in vivo experiments, at least three biological repeats were performed. The sample size was indicated in the main text and figure legends. We did not use statistical methods to predetermine the sample size since the lung phenotypes analyzed in this study are completely penetrant.
Data exclusions	No data were excluded in this study.
Replication	The replication numbers were included in the corresponding figure legends.
Randomization	The control and mutant mouse lungs that we reported in this study were based on the genotyping results, and both sexes were included in all time points.
Blinding	Blinding was irrelevant for mouse work in this study since the experimental groups were assigned based on the genotyping results.<br>For RNA-seq and multiomics library preparation and sequencing, the investigators were blinded to the processing conditions.

Reporting for specific materials, systems and methods

We require information from authors about some types of materials, experimental systems and methods used in many studies. Here, indicate whether each material, system or method listed is relevant to your study. If you are not sure if a list item applies to your research, read the appropriate section before selecting a response.

Materials & experimental systems

n/a	Involved in the study	
[ ]	[x]	Antibodies
[x]	[ ]	Eukaryotic cell lines
[x]	[ ]	Palaeontology and archaeology
[ ]	[x]	Animals and other organisms
[x]	[ ]	Clinical data
[x]	[ ]	Dual use research of concern
[x]	[ ]	Plants

Methods

n/a	Involved in the study	
[x]	[ ]	ChIP-seq
[x]	[ ]	Flow cytometry
[x]	[ ]	MRI-based neuroimaging

Antibodies

Antibodies used	The primary antibodies used for whole-mount immunofluorescence include:<br>rat anti-E Cadherin (1:200, Life Technologies, Cat# 13-1900; RRID:AB_2533005),<br>rabbit anti-phospho-YAP (Ser127) (1:100, Cell Signaling, Cat# 4911S; RRID:AB_2218913),

rabbit anti-SOX2 (D9B8N) (1:200, Cell Signaling Technology, Cat# 23064S; RRID:AB_2714146),
goat anti-SOX9 (1:200, R&D Systems, Cat# AF3075; RRID:AB_2194160).

The primary antibodies used for immunofluorescence and immunohistochemistry include:
rabbit anti-NKX2.1 (1:100, Epitomics, Cat# 2044-1; RRID:AB_1310784),
chicken anti-GFP (1:200, Aves Labs, Cat# GFP-1010; RRID:AB_2307313),
rabbit anti-prosurfactant protein C (proSP-C) (1:200, Seven Hills Bioreagents, Cat# WRAB-9337; RRID:AB_2335890),
Syrian hamster anti-T1a (1:200, Developmental Studies Hybridoma Bank, Cat# 8.1.1; RRID:AB_531893),
mouse anti-HOPX (E-1) (1:100, Santa Cruz Biotechnology, Cat# sc-398703; RRID:AB_2687966),
goat anti-CC10 (1:200, Santa Cruz Biotechnology, Cat# sc-9773; RRID:AB_2183391),
rabbit anti-CCSP (1:200, Seven Hills Bioreagents, Cat# WRAB-3950; RRID:AB_451716),
mouse anti-acetylated tubulin (1:200, Santa Cruz Biotechnology, Cat# sc-23950; RRID:AB_628409),
mouse anti-α-actin (ACTA2) (SMA), clone 1A4 (1:200, Santa Cruz Biotechnology, Cat# sc-32251; RRID:AB_262054),
rat anti-PECAM-1 (CD31) (1:150, Santa Cruz Biotechnology, Cat# sc-18916; RRID:AB_627028),
rabbit anti-PDGFRA (1:150, Cell Signaling Technology, Cat# 3164S; RRID:AB_2162351),
goat anti-PDGFRB (1:200, R&D Systems, Cat# AF1042; RRID:AB_2162633),
rabbit anti-LATS1 (C66B5) (1:100, Cell Signaling Technology, Cat# 3477S; RRID:AB_2133513),
rabbit anti-Phospho-YAP (Ser127) (1:100, Cell Signaling Technology, Cat# 4911S; RRID:AB_2218913),
mouse anti-YAP (63.7) (1:100, Santa Cruz Biotechnology, Cat# sc-101199; RRID:AB_1131430),
rabbit anti-YAP1 (1:100, Novus Biologicals, Cat# NB110-58358; RRID:AB_1849483),
goat anti-CTGF (1:100; Santa Cruz Biotechnology #sc-14939; RRID:AB_638805).

Secondary antibodies and conjugates used include:
donkey anti-rabbit Alexa Fluor® 488 (1:1000, Life Technologies, Cat# A-21206; RRID:AB_2535792),
donkey anti-rabbit Alexa Fluor® 594 (1:1000, Life Technologies, Cat# A-21207; RRID:AB_141637),
donkey anti-chicken Alexa Fluor® 488 (1:1000, Jackson ImmunoResearch, Cat# 703-546-155; RRID:AB_2340376)
donkey anti-chicken Alexa Fluor® 647 (1:1000, Jackson ImmunoResearch, Cat# 703-606-155; RRID:AB_2340380),
donkey anti-mouse Alexa Fluor® 488 (1:1000, Life Technologies, Cat# A-21202; RRID:AB_141607)
donkey anti-mouse Alexa Fluor® 594 (1:1000, Life Technologies, Cat# A-21207; RRID:AB_141637),
donkey anti-rat Alexa Fluor 594 (1:1000, Life Technologies, Cat# A-21209; RRID:AB_2535795).

Biotinylated secondary antibodies used include:
goat anti-hamster (1:1000, Vector Laboratories, Cat# BA-9100; RRID:AB_2336137),
donkey anti-rabbit (1:1000, Jackson ImmunoResearch, Cat# 703-066-155; RRID:AB_2340355),
donkey anti-rat (1:1000, Jackson ImmunoResearch, Cat# 712-065-150; RRID:AB_2340646),
horse anti-mouse (1:1000, Vector Laboratories, Cat# BA-2000; RRID:AB_2313581),
streptavidin-conjugated Alexa Fluor® 488 (1:1000, Jackson ImmunoResearch, Cat# 016-540-084; RRID:AB_2337249),
streptavidin-conjugated Alexa Fluor® 594 (1:1000, Jackson ImmunoResearch, Cat# 016-580-084; RRID:AB_2337250),
streptavidin-conjugated Alexa Fluor® 647 (1:1000, Jackson ImmunoResearch, Cat# 016-600-084; RRID:AB_2341101),
HRP-conjugated streptavidin (1:1000, Jackson ImmunoResearch, Cat# 016-030-084; RRID:AB_2337238) coupled with fluorogenic substrate Alexa Fluor® 594 tyramide (1:200, TSA kit; Akoya Biosciences, Cat# NEL753001KT) or Cyanine 555 (1:200, Biotium, Cat# 96020) for 30 s.

Validation

All the antibodies in this study were purchased from commercial vendors. They have been widely used in mouse lungs and other tissues in publications from our lab and other groups.
e.g., Lin et al., 2017, eLife; Zhang et al., 2020, eLife; Zhang et al., 2022, Developmental Cell; Zhang et al., 2022, PLOS Biology.

Animals and other research organisms

Policy information about studies involving animals; ARRIVE guidelines recommended for reporting animal research, and Sex and Gender in Research

Laboratory animals

The mouse strains used in this study and their sources are listed below.
Yap floxed allele (Yapf) [Yap1tm1.1Eno; MGI: 5446483; Dr. Eric Olson],
Taz floxed allele (Tazf) [Wwtr1tm1.1Eno; MGI: 5544289; Dr. Eric Olson],
Lats1 floxed allele (Lats1f) [Lats1tm1.1Jfm/RjoJ; MGI: 5568586 RRID: IMSR_JAX:024941],
Lats2 floxed allele (Lats2f) [Lats2tm1.1Jfm/RjoJ; MGI: 5568589; RRID: IMSR_JAX:025428],
ROSA26mTmG [B6.129(Cg)-Gt(ROSA)26Sortm4(ACTB-tdTomato,-EGFP)Luo)/J; MGI: 3803814; RRID: IMSR_JAX:007676],
ShhCre [B6.Cg-Shhtm1(EGFP/cre)Cjt)/J; MGI: 3619470; RRID: IMSR_JAX:005622],
SpcCre (SftpcCre) [Tg(Sftpc-cre)1Blh; MGI: 3574949; Dr. Brigid Hogan],
Sox9Cre [Sox9tm3(Cre)Crm; MGI: 3608931; Dr. Benoit de Crombrugghe],
Sox9CreER [C57BL/6-Sox9em1(cre/ERT2)Tchn; MGI: 6386787; RRID: IMSR_JAX:035092],
SpcCreER (SftpcCreER) [Sftpctm1.1(cre/ERT2)Ptch; MGI: 5485991; Dr. Pao-Tien Chuang].

Wild animals

No wild animals were used in this study.

Reporting on sex

Sex was not considered in the study design since sex is not a factor in influencing the outcome of lung development in our studies. The findings apply to both sexes.

Field-collected samples	No field-collected samples were used in this study.
Ethics oversight	The mouse experiments in this study were performed following the protocols approved by the Institutional Animal Care and Use Committee (IACUC) of the University of California, San Francisco (UCSF).

Note that full information on the approval of the study protocol must also be provided in the manuscript.

Plants

Seed stocks	*Report on the source of all seed stocks or other plant material used. If applicable, state the seed stock centre and catalogue number. If plant specimens were collected from the field, describe the collection location, date and sampling procedures.*
Novel plant genotypes	*Describe the methods by which all novel plant genotypes were produced. This includes those generated by transgenic approaches, gene editing, chemical/radiation-based mutagenesis and hybridization. For transgenic lines, describe the transformation method, the number of independent lines analyzed and the generation upon which experiments were performed. For gene-edited lines, describe the editor used, the endogenous sequence targeted for editing, the targeting guide RNA sequence (if applicable) and how the editor was applied.*
Authentication	*Describe any authentication procedures for each seed stock used or novel genotype generated. Describe any experiments used to assess the effect of a mutation and, where applicable, how potential secondary effects (e.g. second site T-DNA insertions, mosiacism, off-target gene editing) were examined.*
