# Supplementary material for: Hippo signaling differentially regulates distal progenitor subpopulations and their transitional states to construct the mammalian lungs
Source: Nat Commun. 2026 Apr 3;17:4802. doi: 10.1038/s41467-026-71253-x (PMC13219436; doi:10.1038/s41467-026-71253-x)
Supplement: Supplementary file 4 — Source Data 1 [file 41467_2026_71253_MOESM4_ESM.pdf]

Fig. 1c: Wet weight of lungs (mg)

**14.5 *dpc***	Control	*Lats1* $^{f/f}$; *Lats2* $^{f/f}$; *Sftpc* $^{Cre/+}$
p Value 0.3534	6.7	6.8
	7.5	7.5
	8.1	7.9
	8.3	8
**18.5 *dpc***	Control	*Lats1* $^{f/f}$; *Lats2* $^{f/f}$; *Sftpc* $^{Cre/+}$
p Value 0.0208	36.5	34.5
	34.8	29.2
	35.2	31.5
	33.3	30.6
**18.5 *dpc***	Control	*Lats1* $^{f/f}$; *Lats2* $^{f/f}$; *Shh* $^{Cre/+}$
p Value 0.0005	33.1	5.9
	36.7	7.6
	34.5	7.1

Fig. 2j: Percentage of cell types in the airways and alveoli

**SOX2+ cells**	Control (column 1)	*Lats1* $^{f/f}$; *Lats2* $^{f/f}$; *Sftpc* $^{Cre/+}$ (column 2)	*Lats1* $^{f/f}$; *Lats2* $^{f/f}$; *Yap* $^{f/+}$; *Taz* $^{f/+}$; *Sftpc* $^{Cre/+}$ (column 3)
	1	0.26802721	0.55194805
	0.99854651	0.163511188	0.5754386
	1	0.118568233	0.64583333
**SCGB1A1+ cells**	Control	*Lats1* $^{f/f}$; *Lats2* $^{f/f}$; *Sftpc* $^{Cre/+}$	*Lats1* $^{f/f}$; *Lats2* $^{f/f}$; *Yap* $^{f/+}$; *Taz* $^{f/+}$; *Sftpc* $^{Cre/+}$
	0.67261905	0.0313253	0.29094412
	0.62545455	0.09467456	0.23544974
	0.63543192	0.12600536	0.22113022
**Ac-tub+ cells**	Control	*Lats1* $^{f/f}$; *Lats2* $^{f/f}$; *Sftpc* $^{Cre/+}$	*Lats1* $^{f/f}$; *Lats2* $^{f/f}$; *Yap* $^{f/+}$; *Taz* $^{f/+}$; *Sftpc* $^{Cre/+}$
	0.30654762	0.04819277	0.1849711
	0.36181818	0.08579882	0.14021164
	0.34114202	0.10455764	0.24324324
**SFTPC+ cells**	Control	*Lats1* $^{f/f}$; *Lats2* $^{f/f}$; *Sftpc* $^{Cre/+}$	*Lats1* $^{f/f}$; *Lats2* $^{f/f}$; *Yap* $^{f/+}$; *Taz* $^{f/+}$; *Sftpc* $^{Cre/+}$
	0.44632087	0.067659	0.31407407
	0.45840708	0.05958132	0.40669856
	0.51060359	0.10962963	0.35073409
**HOPX+ cells**	Control	*Lats1* $^{f/f}$; *Lats2* $^{f/f}$; *Sftpc* $^{Cre/+}$	*Lats1* $^{f/f}$; *Lats2* $^{f/f}$; *Yap* $^{f/+}$; *Taz* $^{f/+}$; *Sftpc* $^{Cre/+}$
	0.52834741	0.932341	0.67703704
	0.51858407	0.93880837	0.58054226
	0.46982055	0.88740741	0.63132137
**SFTPC+HOPX+ cells**	Control	*Lats1* $^{f/f}$; *Lats2* $^{f/f}$; *Sftpc* $^{Cre/+}$	*Lats1* $^{f/f}$; *Lats2* $^{f/f}$; *Yap* $^{f/+}$; *Taz* $^{f/+}$; *Sftpc* $^{Cre/+}$
	0.02533172	0	0.00888889
	0.02300885	0.00161031	0.01275917
	0.01957586	0.00296296	0.01794454

p Value (column 1 vs. 2)	p Value (column 1 vs. 3)	p Value (column 2 vs. 3)
<0.0001	0.0002	0.0002

p Value (column 1 vsc 2)	<0.0001	p Value (column 1 vs. 3)	<0.0001	p Value (column 2 vs. 3)	0.0042
p Value (column 1 vs. 2)	0.0004	p Value (column 1 vs. 3)	0.0074	p Value (column 2 vs. 3)	0.0274
p Value (column 1 vs. 2)	<0.0001	p Value (column 1 vs. 3)	0.0207	p Value (column 2 vs. 3)	0.0002
p Value (column 1 vs. 2)	<0.0001	p Value (column 1 vs. 3)	0.0148	p Value (column 2 vs. 3)	0.0002
p Value (column 1 vs. 2)	0.0005	p Value (column 1 vs. 3)	0.0269	p Value (column 2 vs. 3)	0.0105

Fig. 3i: Relative pYAP intensity

	Control	*Lats1* $^{f/f}$ *; Lats2* $^{f/f}$ *; Sftpc* $^{Cre/+}$
**Proximal**		
p Value 0.0117	0.926762799	0.119003917
	1.270610372	0.383103436
	0.802626829	0.196939803

	Control	*Lats1* $^{f/f}$ *; Lats2* $^{f/f}$ *; Sftpc* $^{Cre/+}$
**Distal**		
p Value 0.8723	0.887266586	1.296431922
	0.972810554	0.989707377
	1.13992286	0.827741383

Fig. 3p: Relative distal SOX9+ area per domain or per bud

**Per domain**	Control	*Lats1* $^{f/f}$ *; Lats2* $^{f/f}$ *; Sftpc* $^{Cre/+}$
p Value <0.0001	1.5820255	0.40826464
	1.44054765	0.27436597
	1.05805218	0.26426041
	1.01560883	0.39815908
	0.98023937	0.27840819
	0.80895012	0.24708095
	1.0277355	0.63766087
	0.78621261	0.52296276
	0.88474182	0.36430546
	0.57045889	0.35773684
	1.12171721	0.31680932
	0.98680798	0.15360452
	0.58662779	0.3425785
	0.66191421	0.35672629
	1.06765247	0.56490083
	1.16163418	0.34005211
	0.99893465	0.34561017
	1.19346669	0.42746521
	1.40669402	0.58460667
	1.18588752	0.86705709
	1.15102334	0.48961441
	1.04188329	0.66646171
	1.10049554	0.31478821
	1.21569893	0.21019566
	1.21115143	0.40321186
	1.43852654	0.43959188
	1.37182984	0.47849829
	1.3036173	0.33449405
	1.032283	0.47647718
	1.2369206	0.48759329
	0.70486284	0.43858133
	1.50724435	0.31125126
	0.95901769	0.28699792
	0.70839979	0.39613797

0.74831675	0.35925268
1.05653635	0.37390574
0.74225342	0.16825758
0.8023815	0.27183958
1.49562295	0.17583675
0.67505144	0.15057285
0.77610705	0.21828011
1.2323731	0.13288812
0.9817552	0.40422242
0.83775096	0.23697539
0.74225342	0.20867982
0.90192127	0.22181705
0.87918376	0.34156794
0.81450818	0.66797755
1.10857999	0.32843072
0.82259262	0.3723899
0.65130337	0.23293317
0.55378472	0.62048141
0.83674041	0.15259396
1.13940195	0.42695993
0.65938782	0.69374673
0.97417603	0.11520339
0.75387481	0.1288459
0.51487831	0.19251093
0.95244908	0.347126
0.65736671	0.21929066
0.86099375	0.14450952
0.80389734	0.17533147
0.85796209	0.18291064
0.80036039	0.23798595
0.9726602	0.43959188
1.06259969	0.3425785
0.53711054	0.32742016
0.94133296	0.24910207
1.24197338	0.23849123
1.06310496	0.28245042
1.31624925	0.22636456

	1.41528375	0.32489377
	1.49107545	0.29962987
	1.25207895	0.31781988
	0.87968904	0.20110065
	0.68919923	0.34308378
	1.15961307	0.30923015
	0.82511901	0.25769179
	0.93324851	0.50376219
	1.33848149	0.30973543
	1.57343577	0.30973543
	1.25864756	0.26274457
	0.90646878	0.36228434

**Per bud**	Control	*Lats1* $^{f/f}$ *; Lats2* $^{f/f}$ *; Sftpc* $^{Cre/+}$
p Value <0.0001	0.97435759	0.39720068
	1.34399741	0.34532141
	1.00840336	0.28209354
	1.07487368	0.57877814
	1.41046772	0.36477614
	0.86573536	0.38260964
	1.22889027	0.32910914
	0.95976654	0.33721527
	0.83979573	0.18806236
	0.9808425	0.17671377
	1.18025345	0.15401659
	0.93706936	0.46691345
	1.28887568	0.25777514
	1.46558945	0.24318409
	1.48180172	0.13780432
	1.42992245	0.18644114
	1.04082791	0.29506336
	1.2661785	0.18644114
	0.98570618	0.36963982
	1.25158745	0.29182091
	1.29860304	0.22048691
	1.09108595	0.11997082
	0.82844713	0.22372936

1.27914832	0.3745035
0.66146073	0.26101759
1.34561863	0.23994164
0.72468859	0.26912373
1.41046772	0.17022886
0.65659704	0.3031695
0.891675	0.25615391
1.38290686	0.24318409
1.23537518	0.31776054
0.93382691	0.37126104
0.77818909	0.2675025
1.21916291	0.41503418
1.1948445	0.23669918
1.29049691	0.31776054
0.86411413	0.23021427
1.29536059	0.2318355
1.23699641	0.32910914
0.98894863	0.27560864
1.04731282	0.21562323
1.01326704	0.60471777
1.04407036	0.70037018
1.10729822	0.37612473
1.16241995	0.40206436
1.43640736	0.42638277
0.87059904	0.34207895
1.25645113	0.20913832
1.07163122	0.32262423
1.46883191	0.463671
1.05704018	0.14915291
0.97597882	0.1605015
0.7668405	0.5171715
1.2661785	0.41827664
0.90788727	0.47664082
1.37480072	0.51230782
1.28239077	0.36801859
0.98570618	0.23507795
1.12837418	0.60796023

1.32130022	0.68253668
0.58202059	0.42638277
0.79115891	0.22048691
1.13648032	0.13618309
0.65173336	0.107001
0.9451755	0.30154827
0.84465941	0.20913832
0.48798941	0.18481991
0.83655327	0.37774595
1.284012	0.19941095
0.81871977	0.31613932
0.69226404	0.22210814
0.64686968	0.37288227
0.93869059	0.23832041
0.81223486	0.321003
0.77332541	0.38585209
0.93058445	0.37288227
0.677673	0.28857845
0.891675	0.37288227
0.72793104	0.31451809
1.19160204	0.36477614
0.56418709	0.50258045
0.51230782	0.34370018
0.89005377	0.59823286
0.87059904	0.40368559
0.78305277	0.50258045
0.84465941	0.53338377
0.76521927	0.43124645
0.94841795	0.34694264
1.01326704	0.26101759
1.34886109	0.43611014
1.30670918	0.37612473
0.76359804	0.52527764
0.77332541	0.37774595
1.14945013	0.70199141
0.88843254	0.54797482
0.90626604	0.5528385

0.88032641	0.61282391
1.09919209	0.52041395
0.72630982	0.36477614
0.80412873	0.38423086
0.74252209	0.17022886
0.78305277	0.53986868
0.93706936	0.22210814
0.76521927	0.23832041
0.64524845	0.23507795
0.64362723	0.31776054
0.7668405	0.3031695
1.12675295	0.24318409
0.85114432	0.30479073
0.7311735	0.50258045
0.69226404	0.39395823
1.11864682	0.36801859
1.20457186	0.20751709
1.43154368	0.21886568
1.12513172	0.1605015
0.85925045	0.35018509
0.927342	0.285336
0.95328163	0.24156286
1.02785809	0.38423086

Fig. 3u: Number of branches from LL1 lineage

**Domain branching**	Control	*Lats1* $^{f/f}$ *; Lats2* $^{f/f}$ *; Sftpc* $^{Cre/+}$
p Value 0.017704299	10	4
	9	7
	9	6

**Bifurcation**	Control	*Lats1* $^{f/f}$ *; Lats2* $^{f/f}$ *; Sftpc* $^{Cre/+}$
p Value 0.001040913	28	10
	32	12
	36	8

Fig. 4i: Percentage of SOX2+ domain relative to the sum of Sox9+ and Sox9-Sox2- domains

**14.5 *dpc***	Control	*Lats1* $^{f/f}$ *; Lats2* $^{f/f}$ *; Sftpc* $^{Cre/+}$
p Value 0.027736417	1	0.750954345
	1	0.875901845
	1	0.618166398

**16.5 *dpc***	Control	*Lats1* $^{f/f}$ *; Lats2* $^{f/f}$ *; Sftpc* $^{Cre/+}$
p Value 0.000407198	1	0.429825746
	1	0.395890254
	1	0.558620391

Fig. 4j: Relative length of the SOX9-SOX2- domain

**14.5 *dpc***	Control	*Lats1* $^{f/f}$ *; Lats2* $^{f/f}$ *; Sftpc* $^{Cre/+}$
p Value <0.0001	0.75800322	2.93460697
	1.32029064	1.86698407
	0.82727215	3.25609164
	1.16615879	2.37537055
	1.04129392	2.2296007
	0.90693706	2.94590696
	1.13824782	2.55018136
	0.90196507	2.2242897
	1.26605069	2.97811192
	0.79687518	3.69272318
	0.966036	3.06704283
	1.11881184	2.09128884
	0.78354119	3.1012818
	1.17858878	1.6466343
	0.94287103	1.57137638
	0.83190514	2.57142534
	0.88761408	1.91512202
	1.07654989	1.75703518
	1.20740375	3.32298757
	0.79495418	2.19739573
	0.95349301	2.86737204
	1.11937684	1.81251813
	0.78320219	2.28643964
	1.18627277	2.20790472
	1.31136364	3.20445069
	0.963437	1.70573324
	0.76772121	3.40197448
	1.13384083	1.5499064
	0.82817614	2.86511204
	1.04174592	1.71861522

	Control	*Lats1* $^{f/f}$ *; Lats2* $^{f/f}$ *; Sftpc* $^{Cre/+}$
**16.5 *dpc***		
p Value <0.0001	1.7383902	3.147837747
	1.56640438	4.386203468
	1.36537759	2.243386682
	1.04841292	4.637402208
	0.70963927	4.100426763
	1.33780562	3.325360564
	2.28169364	3.823916049
	2.20767872	3.099247797
	0.94693902	3.17371472
	1.0664929	2.812567094
	1.62663332	2.344521577
	1.28921567	2.756745151
	2.09411384	3.382538505
	1.37340058	2.412999507
	1.53035742	2.867146037
	1.72155322	3.993415873
	1.17474679	4.568924279
	1.29701266	2.219995706
	2.00936392	2.866468038
	1.53973641	2.433452485
	1.20887275	2.516959399
	1.11960284	3.812164061
	2.14959678	2.337402585
	1.20107576	4.511407338
	0.95473601	3.36773552
	1.81138813	2.920481982
	1.02072795	2.998451902
	0.88614508	2.298982624
	1.28424367	4.327104529
	1.96088697	2.333221589

Fig. 4l: Relative transcript levels (13.5 *dpc*)		
Ctgf	Control	*Lats1* $^{f/f}$ *; Lats2* $^{f/f}$ *; Sftpc* $^{Cre/+}$
p Value 0.000658163	0.985762	8.579852
	1.243679	8.725088
	0.876987	6.522474
Ajuba	Control	*Lats1* $^{f/f}$ *; Lats2* $^{f/f}$ *; Sftpc* $^{Cre/+}$
p Value 0.032000085	1.014633	2.128933
	1.327852	1.893618
	0.952212	1.513612
Cyr61	Control	*Lats1* $^{f/f}$ *; Lats2* $^{f/f}$ *; Sftpc* $^{Cre/+}$
p Value 0.041903629	0.892567	4.767714
	1.234238	2.793403
	1.126765	3.521188

Fig. 5g: Percentage of cells in saccules - 18.5 *dpc* (TAM 14.5 *dpc*)		
**SFTPC+ cells**	Control	*Lats1* $^{f/f}$ *; Lats2* $^{f/f}$ *; Sox9* $^{CreER/+}$
p Value 0.000442876	0.45684211	0.01146789
	0.46164109	0.01237624
	0.48813209	0.01298701
**HOPX+ cells**	Control	*Lats1* $^{f/f}$ *; Lats2* $^{f/f}$ *; Sox9* $^{CreER/+}$
p Value 0.000399571	0.53368421	0.98853211
	0.52968646	0.98762376
	0.50361197	0.98701299
**SFTPC+HOPX+ cells**	Control	*Lats1* $^{f/f}$ *; Lats2* $^{f/f}$ *; Sox9* $^{CreER/+}$
p Value 0.001644533	0.00947368	0
	0.00867245	0
	0.00825593	0

Fig. 5h: Percentage of cells in saccules - 18.5 *dpc* (TAM 15.5-16.0 *dpc*)

**SFTPC+ cells**	Control	*Lats1* $^{f/f}$; *Lats2* $^{f/f}$; *Sox9* $^{CreER/+}$
p Value 0.000536585	0.508888114	0.279157667
	0.548769087	0.198039216
	0.513385293	0.20094284

**HOPX+ cells**	Control	*Lats1* $^{f/f}$; *Lats2* $^{f/f}$; *Sox9* $^{CreER/+}$
p Value 0.000534561	0.48658069	0.719762419
	0.44655656	0.801960784
	0.483226025	0.798467885

**SFTPC+HOPX+ cells**	Control	*Lats1* $^{f/f}$; *Lats2* $^{f/f}$; *Sox9* $^{CreER/+}$
p Value 0.002076517	0.004531196	0.001079914
	0.004674353	0
	0.003388682	0.000589275

Fig. 5o: Percentage of cells in saccules - 18.5 *dpc* (TAM 13.5 *dpc*)

**SFTPC+ cells**	Control (column 1)	*Yap* $^{f/f}$; *Taz* $^{f/f}$; *Sox9* $^{CreER/+}$ (cystic) (column 2)	*Yap* $^{f/f}$; *Taz* $^{f/f}$; *Sox9* $^{CreER/+}$ (non-cystic) (column 3)
	0.461728395	0.558105107	0.603305785
	0.412959381	0.493303571	0.658163265
	0.482578397	0.486901536	0.545189504

**HOPX+ cells**	Control	*Yap* $^{f/f}$; *Taz* $^{f/f}$; *Sox9* $^{CreER/+}$ (cystic)	*Yap* $^{f/f}$; *Taz* $^{f/f}$; *Sox9* $^{CreER/+}$ (non-cystic)
	0.533333333	0.398223538	0.395316804
	0.582205029	0.441964286	0.336734694
	0.513937282	0.474254743	0.45335277

**SFTPC+HOPX+ cells**	Control	*Yap* $^{f/f}$; *Taz* $^{f/f}$; *Sox9* $^{CreER/+}$ (cystic)	*Yap* $^{f/f}$; *Taz* $^{f/f}$; *Sox9* $^{CreER/+}$ (non-cystic)
	0.004938272	0.043671355	0.00137741
	0.00483559	0.064732143	0.005102041
	0.003484321	0.038843722	0.001457726

p Value (column 1 vs. 2)	p Value (column 1 vs. 3)	p Value (column 2 vs. 3)
0.2976	0.0151	0.1094

p Value (column 1 vs. 2)	p Value (column 1 vs. 3)	p Value (column 2 vs. 3)
0.0653	0.0163	0.5116

p Value (column 1 vs. 2)	p Value (column 1 vs. 3)	p Value (column 2 vs. 3)
0.0012	0.961	0.001

Fig. 5p: Percentage of cells in saccules - 18.5 *dpc* (TAM 15.5-16.0 *dpc*)

**SFTPC+ cells**	Control	*Yap* $^{f/f}$ *; Taz* $^{f/f}$ *; Sox9* $^{CreER/+}$
p Value 0.002903978	0.51338826	0.672945205
	0.531029357	0.734013971

	0.554203119	0.76459854

HOPX+ cells	Control	*Yap* $^{f/f}$ *; Taz* $^{f/f}$ *; Sox9* $^{CreER/+}$
p Value 0.0028677	0.483522142	0.324058219
	0.465626161	0.263836647
	0.443134272	0.232664234

SFTPC+HOPX+ cells	Control	*Yap* $^{f/f}$ *; Taz* $^{f/f}$ *; Sox9* $^{CreER/+}$
p Value 0.278067953	0.003089598	0.002996575
	0.003344482	0.002149382
	0.002662609	0.002737226

Fig. 5t: Percentage of cells in saccules - 18.5 *dpc* (TAM 13.5 *dpc*)

SFTPC+ cells	Control	*Yap* $^{f/f}$ *; Taz* $^{f/f}$ *; Sox9* $^{CreER/+}$
p Value 0.003697532	0.47482014	0.59259259
	0.50543478	0.65480896

	0.46084724	0.54942529
	0.51706037	0.61988304
	0.50076336	0.51973684
	0.44834711	0.5282392

**HOPX+ cells**	Control	*Yap* $^{f/f}$ *; Taz* $^{f/f}$ *; Sox9* $^{CreER/+}$
p Value 0.005198247	0.50719424	0.38518519
	0.46603261	0.31488801
	0.51476252	0.40229885
	0.45931759	0.36374269
	0.4870229	0.45888158
	0.53305785	0.45348837

**SFTPC+HOPX+ cells**	Control	*Yap* $^{f/f}$ *; Taz* $^{f/f}$ *; Sox9* $^{CreER/+}$
p Value 0.361066444	0.01798561	0.02222222
	0.02853261	0.03030303
	0.02439024	0.04827586
	0.02362205	0.01637427
	0.01221374	0.02138158
	0.01859504	0.01827243

Supplementary Fig. 2j: Percentage of cells in the airway

**SOX2+ cells**	Control (column 1)	*Lats1* $^{f/f}$ *; Lats2* $^{f/f}$ *; Shh* $^{Cre/+}$ (column 2)	*Lats1* $^{f/f}$ *; Lats2* $^{f/f}$ *; Yap* $^{f/+}$ *; Taz* $^{f/+}$ *; Shh* $^{Cre/+}$ (column 3)
	0.99894958	0	1
	1	0	1
	1	0	1

**SCGB1A1+ cells**	Control	*Lats1* $^{f/f}$ *; Lats2* $^{f/f}$ *; Shh* $^{Cre/+}$	*Lats1* $^{f/f}$ *; Lats2* $^{f/f}$ *; Yap* $^{f/+}$ *; Taz* $^{f/+}$ *; Shh* $^{Cre/+}$
	0.5182534	0	0.49956102
	0.53535354	0	0.55427632
	0.5567266	0	0.52337165

**Ac-tub+ cells**	Control	*Lats1* $^{f/f}$ *; Lats2* $^{f/f}$ *; Shh* $^{Cre/+}$	*Lats1* $^{f/f}$ *; Lats2* $^{f/f}$ *; Yap* $^{f/+}$ *; Taz* $^{f/+}$ *; Shh* $^{Cre/+}$
	0.45740873	0	0.47410009
	0.43250689	0	0.43009868
	0.42219467	0	0.45747126

p Value (column 1 vs. 2)	p Value (column 1 vs. 3)	p Value (column 2 vs. 3)
<0.0001	0.4827	<0.0001

p Value (column 1 vs. 2)	p Value (column 1 vs. 3)	p Value (column 2 vs. 3)
<0.0001	0.7731	<0.0001

p Value (column 1 vs. 2)	p Value (column 1 vs. 3)	p Value (column 2 vs. 3)
<0.0001	0.4837	<0.0001

Supplementary Fig. 2k: Percentage of cells in the saccules

**SFTPC+ cells**	Control (column 1)	*Lats1* $^{f/f}$ *; Lats2* $^{f/f}$ *; Shh* $^{Cre/+}$ (column 2)	*Lats1* $^{f/f}$ *; Lats2* $^{f/f}$ *; Yap* $^{f/+}$ *; Taz* $^{f/+}$ *; Shh* $^{Cre/+}$ (column 3)
	0.54666667	0	0.51182033

	0.5128694	0	0.52482811
	0.5234375	0	0.52071006
**HOPX+ cells**	Control	*Lats1*$^{f/f}$; *Lats2*$^{f/f}$; *Shh*$^{Cre/+}$	*Lats1*$^{f/f}$; *Lats2*$^{f/f}$; *Yap*$^{f/+}$; *Taz*$^{f/+}$; *Shh*$^{Cre/+}$
	0.43649123	0.93504274	0.47044917
	0.47187798	0.92367906	0.45912911
	0.45507813	0.96607431	0.46252465
**SFTPC+HOPX+ cells**	Control	*Lats1*$^{f/f}$; *Lats2*$^{f/f}$; *Shh*$^{Cre/+}$	*Lats1*$^{f/f}$; *Lats2*$^{f/f}$; *Yap*$^{f/+}$; *Taz*$^{f/+}$; *Shh*$^{Cre/+}$
	0.01684211	0	0.0177305
	0.01525262	0	0.01604278
	0.02148438	0	0.01676529

p Value (column 1 vs. 2)	p Value (column 1 vs. 3)	p Value (column 2 vs. 3)
<0.0001	0.6159	<0.0001

p Value (column 1 vs. 2)	p Value (column 1 vs. 3)	p Value (column 2 vs. 3)
	0.7702	

p Value (column 1 vs. 2)	p Value (column 1 vs. 3)	p Value (column 2 vs. 3)
<0.0001	0.8033	<0.0001

Supplementary Fig. 4m: Relative cell proliferation

**SOX9+ cells**	Control	*Lats1* $^{f/f}$ *; Lats2* $^{f/f}$ *; Sftpc* $^{Cre/+}$
p Value 0.750061074	0.627906977	0.553299492
	0.58029197	0.6038961
	0.6191446	0.64583333
	0.6674938	0.65289256

**SOX9-SOX2- cells**	Control	*Lats1* $^{f/f}$ *; Lats2* $^{f/f}$ *; Sftpc* $^{Cre/+}$
p Value 0.013697769	0.466666667	0.529182879
	0.35135135	0.51449275
	0.43103448	0.53521127
	0.46153846	0.60645161

**SOX2+ cells**	Control	*Lats1* $^{f/f}$ *; Lats2* $^{f/f}$ *; Sftpc* $^{Cre/+}$
p Value 0.043707768	0.161042945	0.232620321
	0.17056075	0.19063545
	0.1827112	0.25106383
	0.15466667	0.18942731
